# Eyes-closed hybrid brain-computer interface employing frontal brain activation

**DOI:** 10.1371/journal.pone.0196359

**Published:** 2018-05-07

**Authors:** Jaeyoung Shin, Klaus-Robert Müller, Han-Jeong Hwang

**Affiliations:** 1 Department of Biomedical Engineering, Hanyang University, Seoul, Korea; 2 Machine Learning Group, Berlin Institute of Technology (TU Berlin), Berlin, Germany; 3 Department of Brain and Cognitive Engineering, Korea University, Seoul, Korea; 4 Max Planck Institute for Informatics, Stuhlsatzenhausweg, Saarbrücken, Germany; 5 Department of Medical IT Convergence Engineering, Kumoh National Institute of Technology, Kumi, Korea; School of Psychology, CHINA

## Abstract

Brain-computer interfaces (BCIs) have been studied extensively in order to establish a non-muscular communication channel mainly for patients with impaired motor functions. However, many limitations remain for BCIs in clinical use. In this study, we propose a hybrid BCI that is based on only frontal brain areas and can be operated in an eyes-closed state for end users with impaired motor and declining visual functions. In our experiment, electroencephalography (EEG) and near-infrared spectroscopy (NIRS) were simultaneously measured while 12 participants performed mental arithmetic (MA) and remained relaxed (baseline state: BL). To evaluate the feasibility of the hybrid BCI, we classified MA- from BL-related brain activation. We then compared classification accuracies using two unimodal BCIs (EEG and NIRS) and the hybrid BCI in an offline mode. The classification accuracy of the hybrid BCI (83.9 ± 10.3%) was shown to be significantly higher than those of unimodal EEG-based (77.3 ± 15.9%) and NIRS-based BCI (75.9 ± 6.3%). The analytical results confirmed performance improvement with the hybrid BCI, particularly for only frontal brain areas. Our study shows that an eyes-closed hybrid BCI approach based on frontal areas could be applied to neurodegenerative patients who lost their motor functions, including oculomotor functions.

## Introduction

Brain-computer interfaces (BCIs) have in the past enabled patients to control external devices directly without the help of muscular movements [1–5]. Thus, many research groups have explored BCI technology and considerably improved the performance of BCI systems [6–12]. Various BCI paradigms based on electroencephalography (EEG) have been introduced to implement BCIs for physically challenged patients. These paradigms include motor imagery [13–15], P300 [16–18], steady-state visual evoked potential (SSVEP) [19], and others. However, these paradigms have limitations with respect to severely motor-impaired patients such as late-stage amyotrophic lateral sclerosis (ALS). For example, some of them cannot generate reliable sensorimotor rhythms for motor-imagery-based BCI [20–22]. Also, as BCIs based on exogenous paradigms such as conventional visual P300 and SSVEP generally require moderate oculomotor functions, these exogenous paradigms cannot be fully exploited for those with oculomotor dysfunctions that are often presented in late-stage ALS or completely locked-in state (CLIS) patients [23]. To overcome these constraints, previous studies introduced BCIs based on cognitive tasks instead of motor imagery tasks and validated its feasibility with both healthy subjects and ALS patients [20–22]. Also, an eyes-closed (EC) SSVEP-based BCI was recently introduced [19], which validated the feasibility of an SSVEP-based BCI under EC conditions for healthy participants and for an (ALS) patient with partially impaired oculomotor functions. A more recent study introduced a novel EC BCI paradigm based on visual P300 and demonstrated its effectiveness [23].

In our previous study, we first proposed an EC BCI system using a representative endogenous BCI paradigm, namely, mental arithmetic (MA), to check whether an endogenous BCI paradigm can also be used in an EC condition [24]. In [24], we used near-infrared spectroscopy (NIRS) signals of prefrontal cortex (PFC) areas, which represents a promising alternative to EEG for BCI research, as its sensitivity to physiological artifacts (e.g., electrooculogram (EOG)) is limited. It has been well documented that EEG signals significantly change under an EC state (e.g., α-rhythm). However, because PFC hemodynamic changes are irrelevant to an EC condition [25], the feasibility of the EC NIRS-BCI could be successfully verified. Moreover, because PFC is essentially below the hair-free region of the skull, we could shorten preparation time and speed up the experiment. In fact, many NIRS-BCI studies have focused on PFC hemodynamic changes, as PFC areas are free from one of the critical drawbacks of a NIRS-based BCI: signal amplitude attenuation as a result of dense, long, and dark hairs blocking light penetration to the scalp [26–32].

Although we successfully demonstrated the feasibility of an EC NIRS-BCI system, the classification accuracy was relatively low compared to those reported in standard EEG-BCI studies [19, 23]. One possible means of improving classification accuracy is to use a hybrid approach that combines two brain-imaging modalities (e.g., EEG and NIRS [33–36]). That the hybrid BCI can increase the reliability of BCI systems in terms of performance has already been demonstrated [37, 38]. In particular, the performance of BCI systems could be enhanced by integrating two kinds of BCI systems or using complementary information of brain activations measured with different modalities. As an example of the former case, a hybrid EEG-NIRS BCI using the SSVEP paradigm was studied, in which NIRS and EEG signals were utilized to operate a brain switch and produce an actual BCI command, respectively [39]. As an example of the latter case, EEG and NIRS data were simultaneously used to decode motor imagery tasks. Here, the decoding performance improved considerably compared to that when using the unimodal data (EEG or NIRS) [33].

Although hybrid EEG-NIRS BCIs have proven useful, they may still be impractical in real clinical scenarios because most hybrid BCI systems employ sensor positions of the scalp with hairs. For NIRS, dense hairs interfere with light penetration to the scalp, which leads to a decrease in signal-to-noise ratio. Regarding EEG, recording EEG signals from the scalp covered in hair also creates practical concerns (attaching electrodes and washing hairs after the experiment). This is particularly the case with patients. Employing frontal areas that include hair-free regions as much as possible is a potential means of reducing this problem. In this case, as a result of the location of their respective brain sources, non-motor tasks (e.g., MA) are considered more appropriate than standard BCI paradigms (e.g., motor imagery). However, no study has yet been conducted to investigate the feasibility of using frontal areas for a hybrid EEG-NIRS BCI approach.

Our previous research aimed to verify the feasibility of the EC NIRS-BCI based on an endogenous BCI paradigm, namely, MA [24]. This study proposes a hybrid EEG-NIRS BCI that utilizes frontal areas, including a hair-free PFC, to improve the BCI performance of our previous EC NIRS-BCI in terms of classification accuracy in an offline mode. To this end, our present study examines the performance of an EC hybrid EEG-NIRS BCI operated by MA that uses only the frontal areas for a convenient system setup.

## Materials and methods

### Participants

Twelve participants participated in the experiment (five males and seven females, 26.7 ± 3.7 (mean ± standard deviation)), none of whom reported any previous or current mental illness. They were given detailed information about the experiment, and a written consent was obtained from each. After completing the experiment, participants were financially reimbursed. Our experiment was performed in compliance with the Declaration of Helsinki and was approved by the Ethics Committee of the Institute of Psychology and Ergonomics, Berlin Institute of Technology (approval number: SH_01_20150330).

### Instrumentation

**Fig 1:**
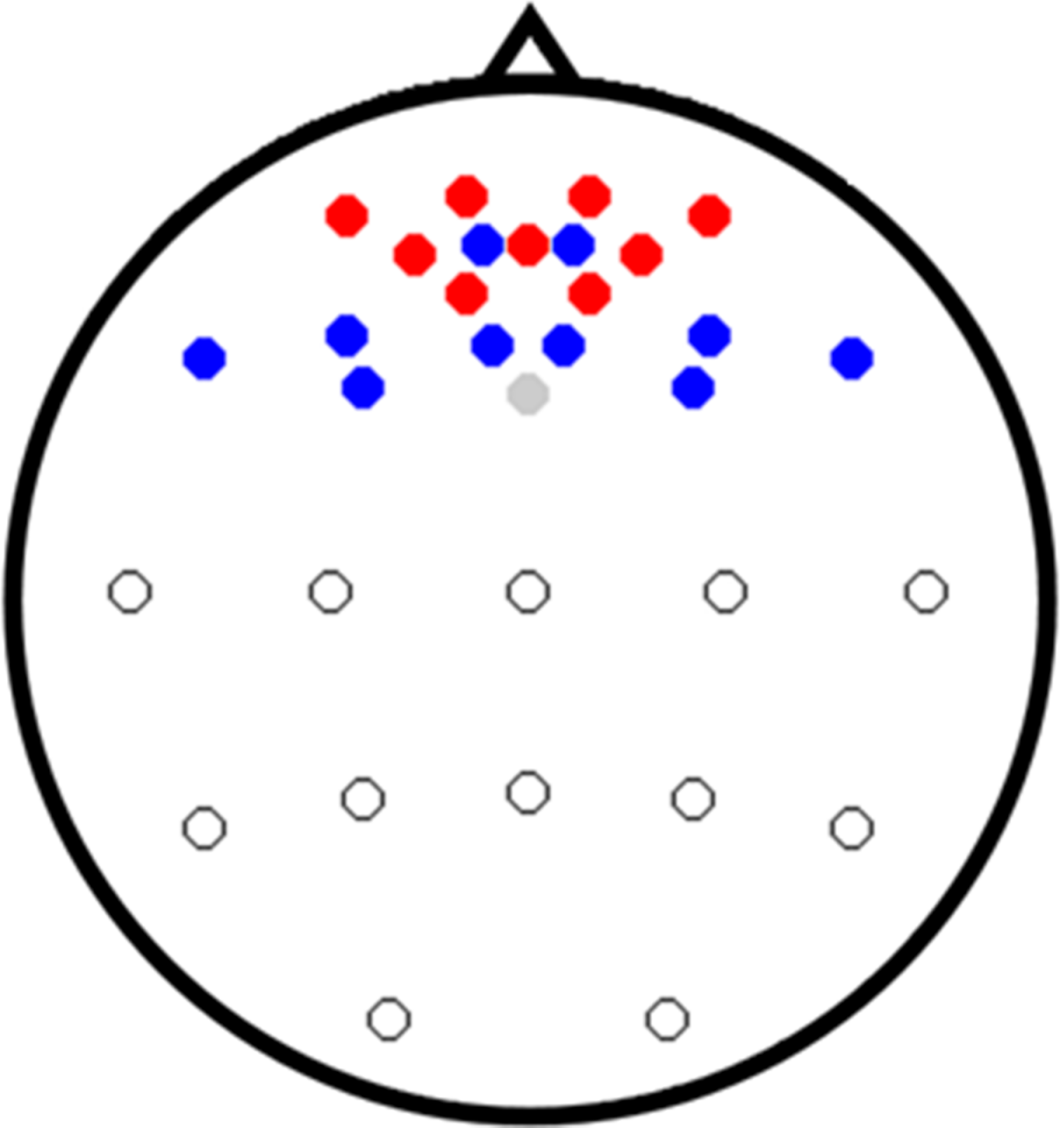
Location of EEG electrodes (blue, white, and gray circles) and NIRS channels (red circles). Gray indicates the ground electrode. Blue and white electrodes represent frontal and parieto-occipital EEG channels, respectively. Note that only the frontal EEG channels were used with NIRS channels in data analysis to investigate the feasibility of the EEG-NIRS EC BCI employing only frontal areas.

A brainAmp amplifier (Brain Products GmbH, Gliching, Germany) was used to record EEG signals using linked mastoid reference (sampling rate: 1000 Hz) from 22 locations on a custom-made elastic cap (EASYCAP GmbH, Herrsching, Germany; AFp1, AFp2, AFF1h, AFF2h, AFF5h, AFF6h, F3, F4, F7, F8, Cz, C3, C4, T7, T8, Pz, P3, P4, P7, P8, OI1, and OI2). The ground electrode was placed on Fz. A NIRScout (NIRx GmbH, Berlin, Germany) was used to record NIRS signals (sampling rate: 12.5 Hz). Five NIR light sources and three detectors were positioned on the PFC. The adjacent sources and detectors consisted of nine channels near Fp1, Fp2, and Fpz. The inter-optode distance was set as 30 mm. NIRS optodes were placed on the same cap as the EEG electrodes. Fig 1 shows the EEG electrodes (blue and white circles) and NIRS channels (red circles). The one gray circle indicates the ground electrode. EOG was recorded using the same BrainAmp amplifier at the same sampling rate of the EEG using two vertical (above and below the left eye) and two horizontal (the outer canthus of each eye) electrodes. All signals were recorded simultaneously, and trigger signals were sent to each system via a parallel port using MATLAB for data synchronization. All data used in this study are fully available without any restriction from the following website: https://doi.org/10.6084/m9.figshare.5900842.v1

### Experimental paradigm

**Fig 2:**
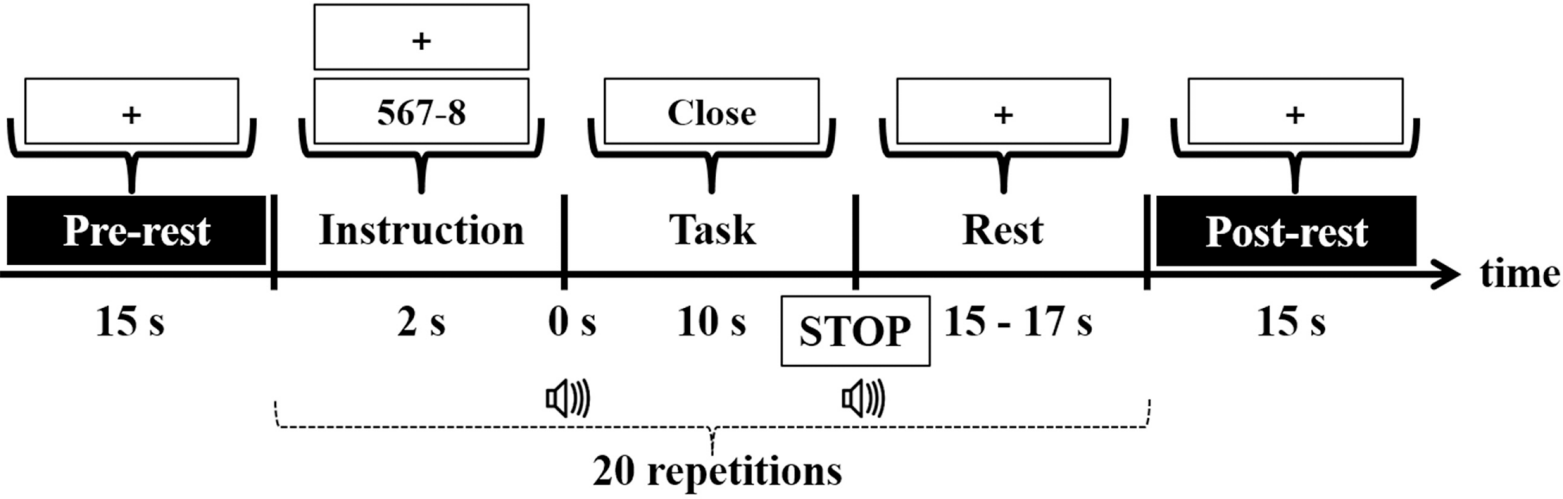
Block of the experimental paradigm. Each session consisted of pre-rest (15 s) staring at a fixation cross, then 20 repetitions of a single trial followed by a post-rest (15 s). With respect to the instruction period, “567–8” and “+” indicate MA and BL, respectively. Note that different combinations of three- and one-digit numbers were used to prevent the participants from becoming accustomed to the problem.

All participants were seated in a comfortable armchair 1.6 m from a 50-inch white screen and all instructions were displayed by a video projector. Fig 2 shows the experimental paradigm. Each session consisted of a pre-rest (15 s) with a fixation cross and 20 repetitions of a single trial followed by a post-rest (15 s). In the pre- and post-rest periods, participants rested with their eyes open while looking at a fixation cross that was displayed in the middle of the screen. A single trial included a visual instruction (2 s) indicating the type of task, a task period (10 s), and a rest period with a random length (15 to 17 s). In the instruction period, the type of task was randomly given (MA or BL). For MA, an arbitrary three-digit number minus a one-digit number between 6 and 9 was given as an initial calculation. For BL, a fixation cross was displayed. Participants were instructed to close their eyes as soon as they recognized the type of task. During the task period that began with a short beep (250 ms), participants were asked to continue performing the given task with their eyes closed. For MA, participants continuously subtracted a one-digit number from the result of their previous calculation. For BL, participants remained relaxed. After the short beep (250 ms), a “STOP” sign was displayed on the screen, the fixation cross reappeared, and participants relaxed with their eyes open while looking at the cross.

## Data analysis

### Preprocessing

**Fig 3:**
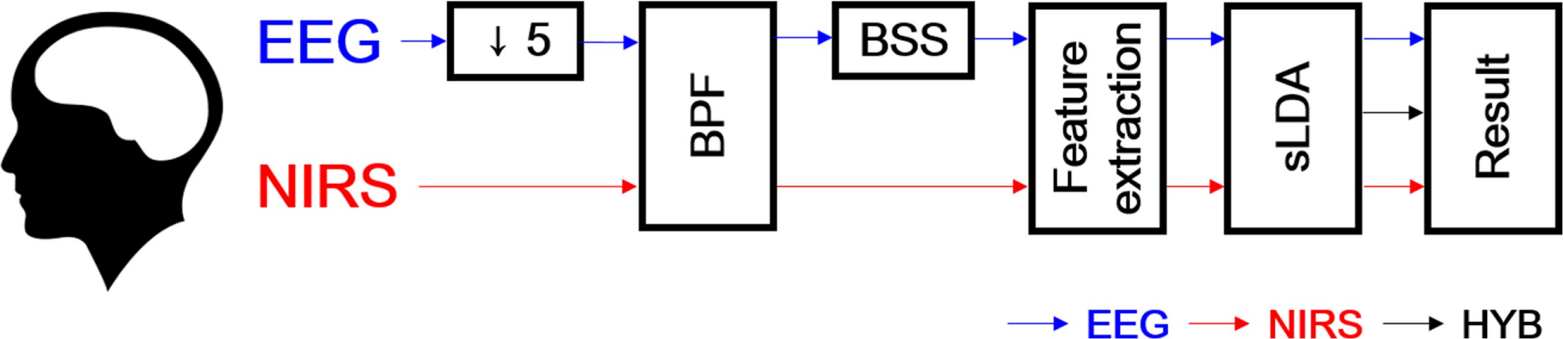
Flow of EEG and NIRS data processing and analysis. EEG data were downsampled by 5. Both EEG and NIRS data were band-pass filtered (BPF). Blind source separation (BSS) was performed to remove ocular artifacts in the EEG data. For both sets of data, feature vectors were independently constructed and shrinkage linear discriminant analysis (sLDA) was used to discriminate between specific task-related brain activations. For the hybrid approach, the classifier outputs of EEG and NIRS data were concatenated, thus creating a separate feature vector. The classifications of EEG, NIRS, and hybrid data were performed separately.

MATLAB R2013b (MathWorks, Natick, MA, USA) was used for data analysis. For data processing and analysis, only frontal EEG electrodes (AFp1, AFp2, AFF1h, AFF2h, AFF5h, AFF6h, F3, F4, F7, and F8) were used together with the NIRS channels in order to investigate the feasibility of the EC hybrid BCI employing only frontal areas. The EEG signals were downsampled to 200 Hz and band-pass filtered (3rd-order Butterworth filter with 0.5–50 Hz passband) before EOG rejection. Blind source separation-based EOG rejection was performed using the automatic artifact rejection toolbox in EEGLAB [40, 41]. For NIRS, the modified Beer-Lambert law was applied to convert light intensity changes to the concentration changes of deoxy- and oxyhemoglobin (ΔHbR and ΔHbO) [42]. ΔHbR and ΔHbO were band-pass filtered using a 6th-order zero-phase Butterworth filter with a passband of 0.01–0.2 Hz. Fig 3 provides a flow of the data processing and analytical procedure.

### Feature extraction

We used EEG epochs acquired from the entire task period (i.e., 0–10 s) and NIRS epochs acquired from the end of the task period for 5 s (i.e., 10–15 s) based on the preliminary analysis investigating the impact of analysis time window on classification performance (see S1 Fig). For EEG, prior to spatial filtering, the EEG data were band-pass filtered with a participant-specific passband. For 0–10 s EEG data, the participant-specific passband was selected using the heuristic band selection method based on signed *r*^2^-values (sgn *r*^2^) using point biserial correlation coefficient with sign preserved [43, 44]. The common spatial pattern (CSP) filter was then applied to the filtered EEG data [45–50]. Note that the CSP filter and participant-specific passband were determined based only on the training data within the inner cross-validation loop to avoid over-fitting. Feature vectors were produced by calculating the log variances of the first and last three CSP components based on the ratio-of-medians score. This score is more robust with respect to outliers than conventional eigenvalue scores [47]. For NIRS data, considering the hemodynamic delay, the mean value and average slope of ΔHbR and ΔHbO between 10 and 15 s were used to create feature vectors, as the hemodynamic change fully developed during the period [51] and showed the highest discriminative information (see the result section for details).

### Classification

A shrinkage linear discriminant analysis (sLDA) was used as a classifier [48, 52]. The classification performance was calculated by 10 x 5-fold cross-validation. The same classifier and cross-validation approach were applied to EEG and NIRS. The combined EEG and NIRS data were evaluated according to a meta-classification method. The outputs of each classifier (i.e., EEG, HbR, and HbO) were combined to build new feature vectors for the meta-classifier [33]. The classification performance of all possible combinations of EEG and both NIRS chromophores were examined (HbR+HbO, EEG+HbR, EEG+HbO, and EEG+HbR+HbO).

### Information transfer rate (ITR)

Among diverse metrics to assess the performance of a BCI system, information transfer rate (ITR), in bits per minute, has been commonly used as a measure of the BCI performance [53]. ITR can be computed as [7]

$$ITR=m*(\log_{2}N+P\log_{2}P+(1-P)\log_{2}\frac{1-P}{N-1})$$ (1)

where *m* is number of trials per minute, *N* is the number of task types and *P* is the classification accuracy (*N* is 2 in this study). Because the length of the rest period (15–17 s) was redundantly long in terms of unimodal EEG-BCI, in order to fairly assess the ITR, we set a single trial length considering only the length of the task period (10 s) excluding the length of the rest period.

## Experimental results

### Spectral, temporal, and spatial characteristics

**Fig 4:**
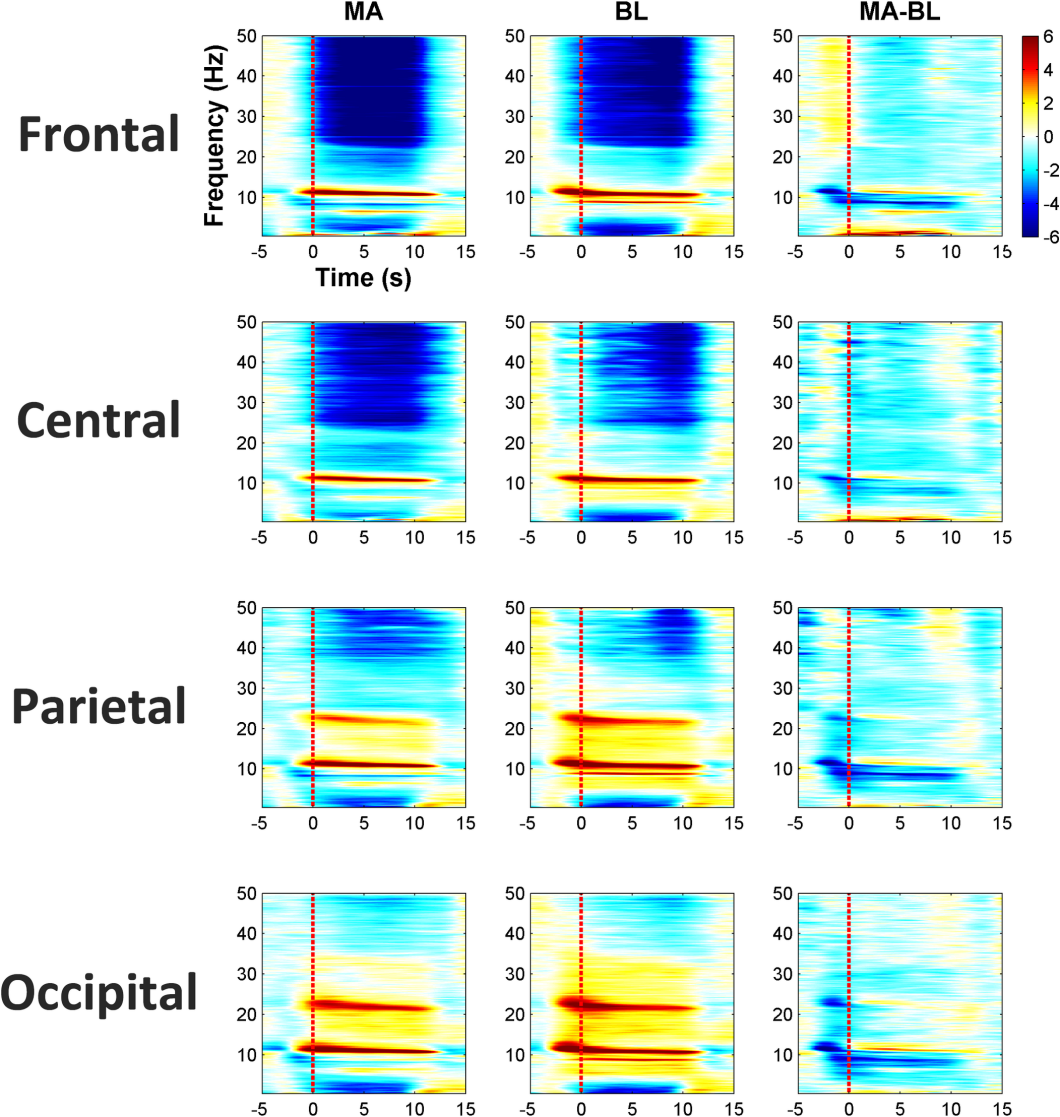
Grand average of time-frequency analysis results for MA, BL, and the difference between MA, BL (MA-BL). The spectral power changes were averaged over 10 frontal (AFp1, AFp2, AFF1h, AFF2h, AFF5h, AFF6h, F3, F4, F7, and F8), five central (Cz, C3, C4, T7, and T8), five parietal (Pz, P3, P4, P7, and P8), and two occipital channels (POO1 and POO2). The colorbar indicates the spectral power in dB.

Fig 4 shows the grand-average time-frequency analysis results of EEG, in which spectral power changes due to MA and BL and the difference between MA and BL are shown. The spectral power changes were averaged over 10 frontal channels (AFp1, AFp2, AFF1h, AFF2h, AFF5h, AFF6h, F3, F4, F7, and F8). For reference, mean spectral power changes of five central (Cz, C3, C4, T7, and T8), five parietal (Pz, P3, P4, P7, and P8), and two occipital channels (POO1 and POO2) were also provided. Note that only the 10 frontal electrodes were used for classification. An increase of natural α-rhythm power with closed eyes was observed over whole areas. The second harmonic of the natural α-rhythm was clearly observed in the occipital area. By contrast, decrease in power resulting from closed eyes was clearly observed except in the α- and low β-bands in the frontal and central areas [54]. In accordance with [55], a decrease in task-related α-rhythm spectral power was also observed through whole brain areas, and thus distinct spectral power difference between MA and BL (MA-BL) was observed (see the third column in Fig 4).

**Fig 5:**
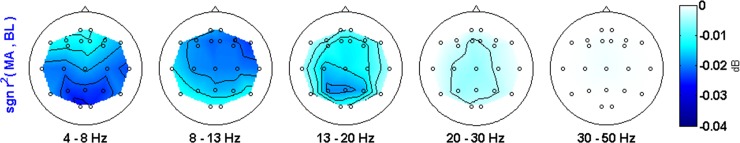
Signed r^2^-values (sgn r^2^) in the θ- (4–8 Hz), α- (8–13 Hz), low β- (13–20 Hz), high β- (20–30 Hz), and γ-band (30–50 Hz). The colorbar indicates the level of sgn r^2^ ranging from -0.04 to 0 dB. Note that the lower value (dark blue) indicates better separability than the higher value (light blue). Considering only frontal areas used for data analysis, the highest separability is shown in the α-band; moderate separability is shown in the θ- and low β-band.

Fig 5 shows task-related spectral power difference in terms of signed r^2^-values (sgn r^2^) for each frequency band in detail. Dark blue indicates the higher separability of MA and BL. In the θ- (4–8 Hz) and low β-band (13–20 Hz), parietal and occipital areas showed higher separability. In addition, higher separability was apparent near frontal areas in the α-band (8–13 Hz). The high β- (20–30 Hz) and γ-bands (30–50 Hz) do not present a strong and meaningful spectral power difference between MA and BL for discrimination. Considering only frontal areas were used for classification, the highest separability appeared in the α-band, whereas moderate separability was apparent in the θ- and low β-band. Conforming to the results shown in Figs 4 and 5, the α-band was mostly included in participant-specific passbands for classification, followed by the θ- and low β-band (see Table 1).

**Table 1:** Individual classification accuracy of EEG, NIRS, and hybrid approaches (HYB). Note that “std” refers to standard deviation and [*f*_L_ *f*_H_] indicates the most frequently selected participant-specific passbands for CSP filters estimated by the heuristic method. The p-values were corrected by the false discovery rate.

Participant	Classification Accuracy			
	EEG	[*f*_L_ *f*_H_]	NIRS	HYB
**1**	96.5	[4.0 11.5]	76.0	96.3
**2**	79.0	[5.5 10.5]	75.3	**84.0**
**3**	58.2	[14.0 35.0]	67.3	**67.5**
**4**	90.7	[4.5 10.5]	81.0	**92.3**
**5**	95.7	[8.0 12.5]	74.5	**96.2**
**6**	83.0	[14.5 19.5]	89.5	**91.8**
**7**	50.7	[23.5 35.0]	71.8	**70.2**
**8**	66.2	[19.5 23.5]	71.8	**74.2**
**9**	81.2	[4.0 8.0]	68.0	**85.7**
**10**	76.8	[24.5 35.0]	77.8	**80.2**
**11**	96.0	[4.0 10.5]	85.0	95.3
**12**	53.7	[6.0 9.0]	73.0	**72.8**
**mean**	77.3		75.9	83.9*^,^^†^
**std**	15.9		6.3	10.3

Wilcoxon signed rank sum test

*p < 0.01 (NIRS vs. HYB) and

^†^*p* < 0.01 (EEG vs. HYB)

p-values are corrected by the false discovery rate

**Fig 6:**
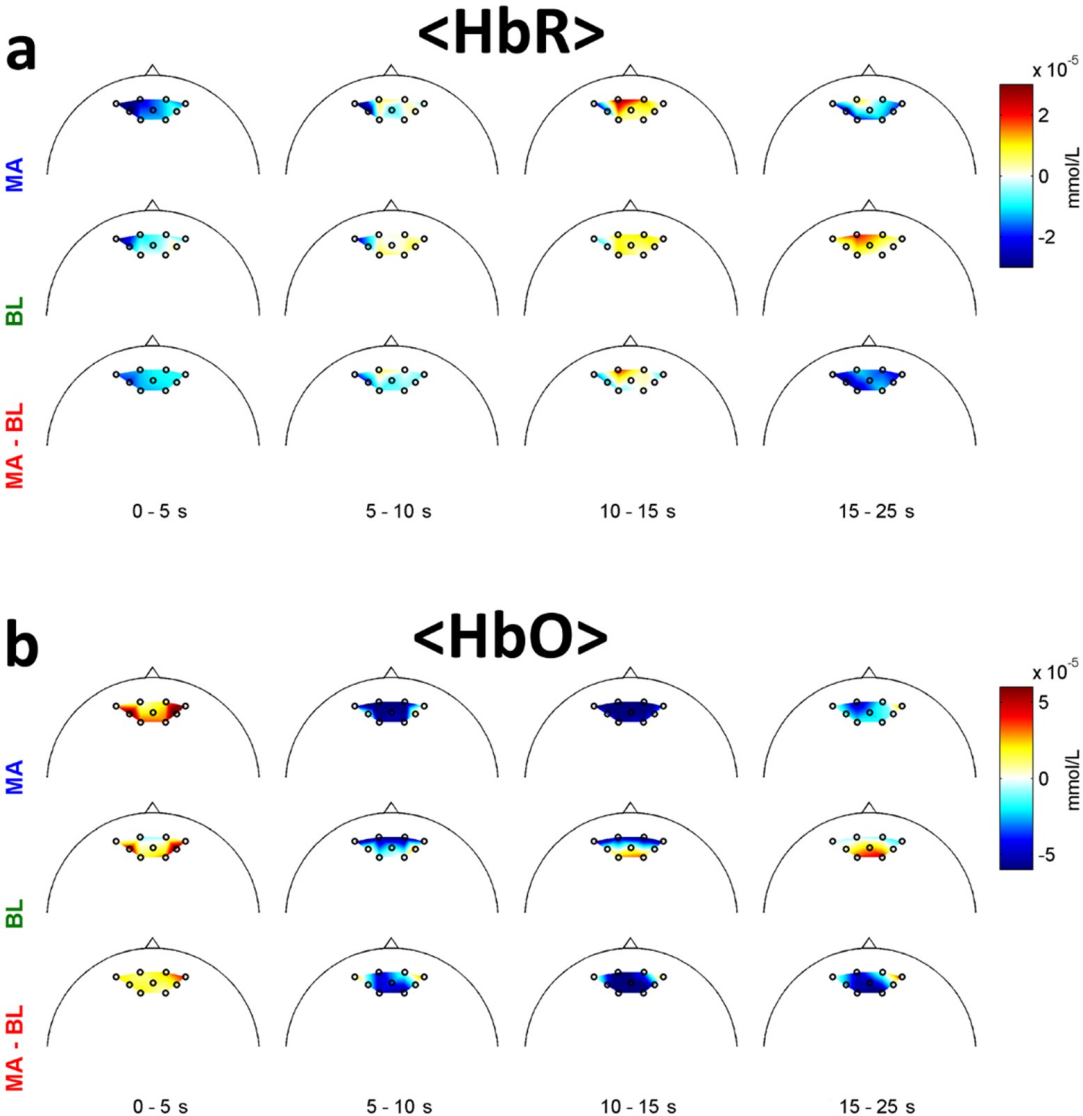
Spatial distribution of hemodynamic responses at given time intervals. (a) Δ[HbR] for MA and BL. (b) Δ[HbO] for MA and BL. The colorbar indicates the amount of change in concentration.

Fig 6 shows the grand average scalp maps of hemodynamic responses. HbR decrease and HbO increase were observed in the early stage (0–5 s) for both MA and BL. After 5 s, an opposite trend was observed, and they peaked at 10–15 s. For HbR, the activation was stronger at anterior channels than posterior ones. Then, the amplitude of activation generally decreased. Note that even though MA and BL induced similar spatial patterns of hemodynamic responses for each period, MA led to higher brain activation than BL in general.

### Performance

**Fig 7:**
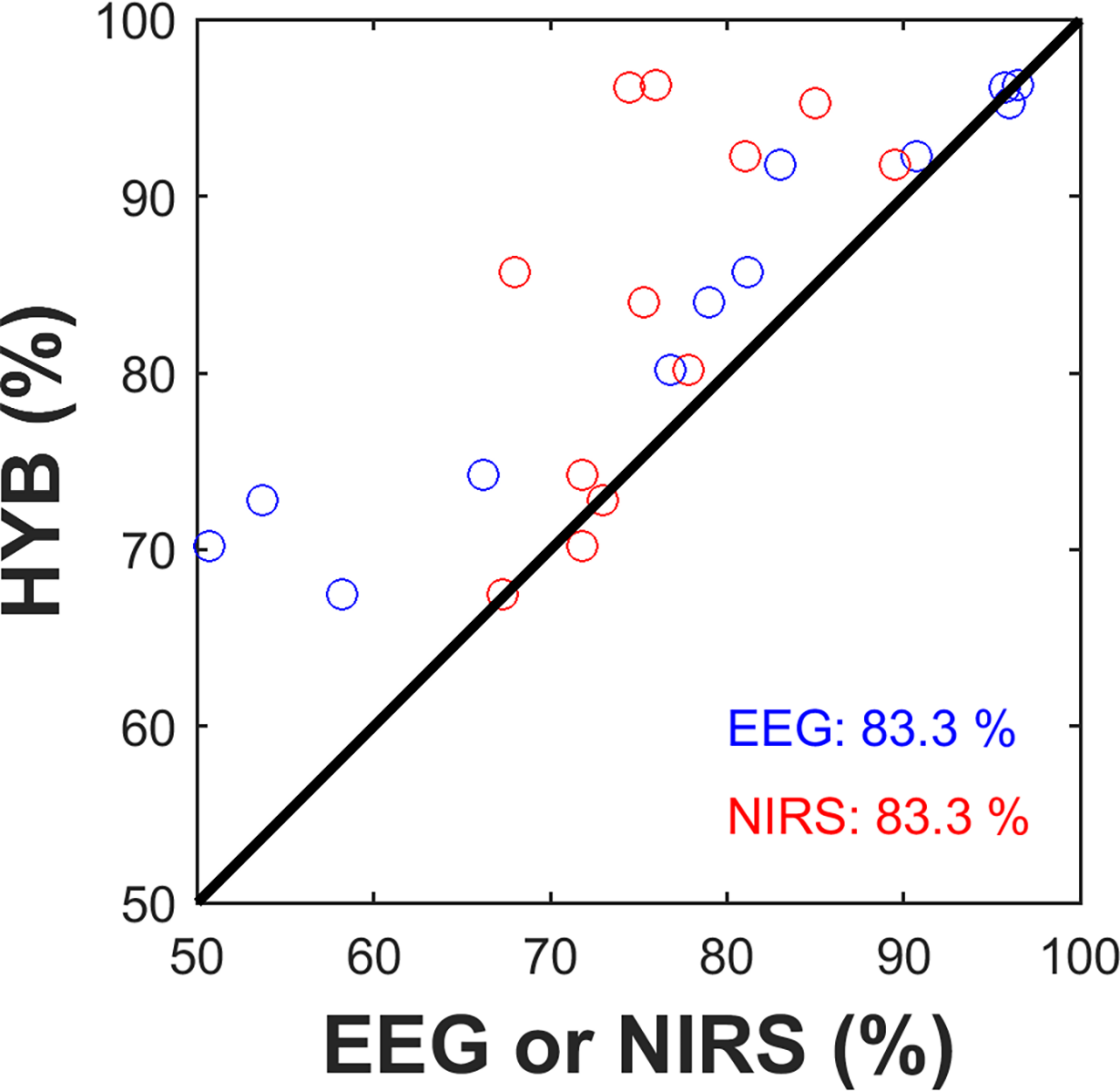
Comparison of classification accuracies of unimodal BCI (EEG or NIRS) and the HYB. Each circle indicates the individual result. Blue and red circles refer to the performance of EEG vs. HYB and NIRS vs. HYB, respectively. Note that the circles above the diagonal line indicate that the individual classification accuracy improved by HYB. Percentage values show the ratio of the number of improved individual classification accuracies by HYB over the total number of participants.

The offline classification accuracies of EEG, NIRS, and hybrid approach and their averages are shown in Table 1. Frontal channel EEG scored 77.3 ± 15.9% and NIRS reached a slightly lower classification accuracy (75.9 ± 6.3%), but the difference was not statistically significant (Friedman test, p = 0.018; post-hoc: Wilcoxon signed rank sum test with false discovery rate (FDR) correction, corrected-p = 0.694). The average hybrid approach (HYB) had significantly higher classification accuracy (83.9 ± 10.3%) than those derived from EEG or NIRS. Most participants (10 of 12) showed considerably improved classification accuracies when using the hybrid approach (bold numbers in Table 1). Performance improvement with respect to classification accuracy is shown in Fig 7. Blue and red circles indicate individual performances for unimodal EEG or NIRS, respectively, compared to HYB. 83.3% of participants showed improved classification accuracies by HYB (FDR corrected-p < 0.01 for both EEG vs. HYB and NIRS vs. HYB).

**Fig 8:**
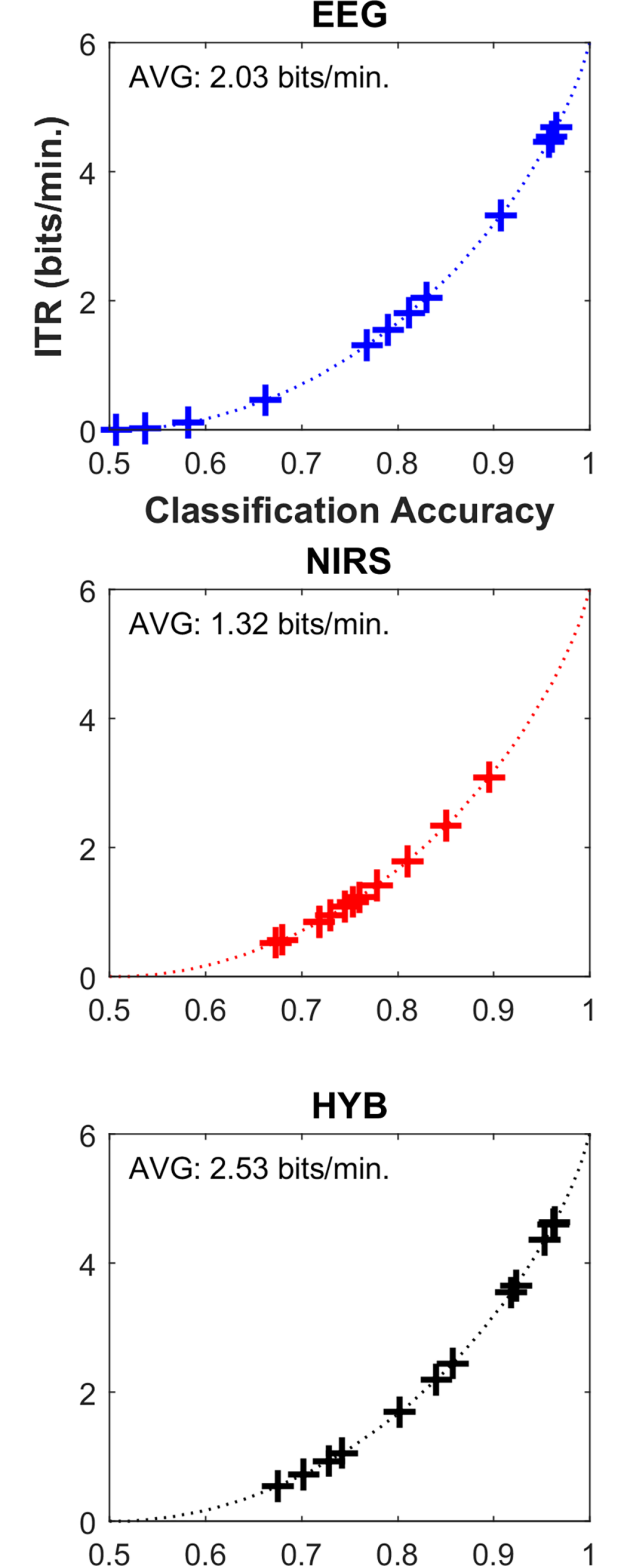
Information transfer rates (ITRs) computed by using EEG (top), NIRS (middle) and HYB (bottom). Average ITRs (AVG) are shown in the corresponding subfigures. The ITRs of NIRS are obtained using the classification accuracies of HbR+HbO. Dotted lines are theoretical ITRs according to classification accuracy. ‘+’ symbols indicate the individual ITRs.

Fig 8 shows the average ITRs across all participants. NIRS scores lower ITR (1.32 bits/min.) than EEG (2.03 bits/min.) while HYB shows ITR improvement (2.53 bits/min.). Note that significant difference is verified between EEG/NIRS and HYB (Friedman test: p = 0.018, post-hoc: Wilcoxon signed rank sum test with false discovery rate-corrected p < 0.01), while no significant difference between EEG and NIRS (corrected p = 0.301).

## Discussion

In our previous EC NIRS-based BCI study [24], we confirmed that the performance of an EC NIRS-based BCI (75.6 ± 7.3%) was comparable with that of an EO NIRS-based BCI (77.0 ± 9.2%), thus demonstrating for the first time the feasibility of an EC NIRS-based BCI. In this study, we attempted to implement a hybrid EEG-NIRS BCI using only frontal brain activations that were generated by MA and BL under an EC state in order to improve the performance of an EC NIRS-based BCI. As expected, we confirmed in our offline study that the classification accuracy of an EC NIRS-based BCI as implemented in this study was similar to the previous one (76.4 ± 6.3%), even though participants differed between the two studies. With the hybrid EEG-NIRS BCI, we achieved significantly improved classification accuracy (83.9 ± 10.3%) compared to that obtained using only NIRS (75.9 ± 6.3%) or EEG (77.3 ± 15.9%). It is noteworthy that the performance improvement was accomplished using only a few frontal EEG electrodes, and not all EEG electrodes were attached over the entire scalp.

For EEG, the α-rhythm induced under an EC state appeared over whole scalp areas (Fig 4). Nevertheless, EEG classification accuracy was not considerably affected by the natural α-rhythm. The decrease in α-power during the MA task was still detectable when eyes were closed, as shown in Fig 4. Based on this neurophysiological phenomenon, despite the presence of very strong α-rhythms, we were able to achieve reasonable classification accuracy using only the frontal EEG, which is comparable to that of a previous study in which 75.9% classification accuracy was achieved and which was estimated by using EEG electrodes distributed over the entire scalp [44].

As CLIS patients are generally bed-ridden and artificially ventilated through tracheostoma, sensors used to capture brain activity in an experiment should be carefully attached. In fact, a recent EEG-based BCI study performed with ALS patients reported the same difficulty, where EEG electrodes were attached around occipital areas to measure SSVEPs [56]. As a result, our hybrid BCI employing only frontal brain areas is expected to provide a safer and more convenient means of communicating with paralyzed patients, especially those having impaired oculomotor functions.

In most BCI studies, BCI systems were designed and assessed in eyes-open state, while a few number of recent BCI studies were conducted for verifying the feasibility of EC BCI systems [23, 24, 57]. Our results added another piece of evidence to previous EC BCI studies, demonstrating its feasibility. However, BCIs that are independent of the state of the eyes should be fundamentally developed for end users such as late-state ALS patients because they are frequently unable to voluntarily control their eye-lids, e.g., opening and closing the eyes. To develop a BCI system independent of eyes’ state, one study investigated the effect of the state of the eyes on BCI performance, and showed closing the eyes during a cognitive task decreases BCI performance compared to eyes-open condition for amplitude modulation but not for frequency modulation features [58]. This result can be utilized when developing a hybrid BCI system totally independent of eyes’ state in future studies.

Recently, one BCI study was the first to apply simultaneous recordings of EEG and NIRS for ALS and CLIS patients. However, the modalities were independently employed and not fused for data analysis [59]. In addition, the locations of EEG electrodes and NIRS optodes were somewhat different from that (i.e., the brain region) used in our study, as the fronto-central region was used in [59]. Thus, further studies for patients having neurodegenerative diseases must be conducted in order to address thoroughly the clinical benefits of EEG and NIRS hybrid BCIs when employing only frontal brain areas.

## Conclusion

In this study, we proposed an EC hybrid BCI that combines EEG and NIRS in order to improve the performance of the single modality-based EC BCI. Specifically, only frontal brain areas were used to discriminate MA-related brain activation from that related to BL. We achieved a promising classification accuracy with our hybrid BCI under EC condition. Our results provide evidence that a hybrid EEG-NIRS BCI can be implemented with only frontal areas and EC, and may be useful for future applications in studies on end users having oculomotor dysfunctions.

## Supporting information

S1 Fig Impact of analysis time window on classification performance. EEG, NIRS and HYB classification accuracies calculated by using various time windows. The Tables below the figures denote time periods for EEG and NIRS data used for calculating the corresponding classification accuracies. For the upper panel, the EEG time window is fixed and the NIRS time window varies, while the EEG time window varies and the NIRS time window is fixed for the lower panel.
(DOCX)
