# Supplementary material for: Eyes-closed hybrid brain-computer interface employing frontal brain activation
Source: PLoS One. 2018 May 7;13(5):e0196359. doi: 10.1371/journal.pone.0196359 (PMC5937739; doi:10.1371/journal.pone.0196359)
Supplement: S1 Fig — EEG, NIRS and HYB classification accuracies calculated by using various time windows. The Tables below the figures denote time periods for EEG and NIRS data used for calculating the corresponding classification accuracies. For the upper panel, the EEG time window is fixed and the NIRS time window varies, while the EEG time window varies and the NIRS time window is fixed for the lower panel. (DOCX) [file pone.0196359.s001.docx]

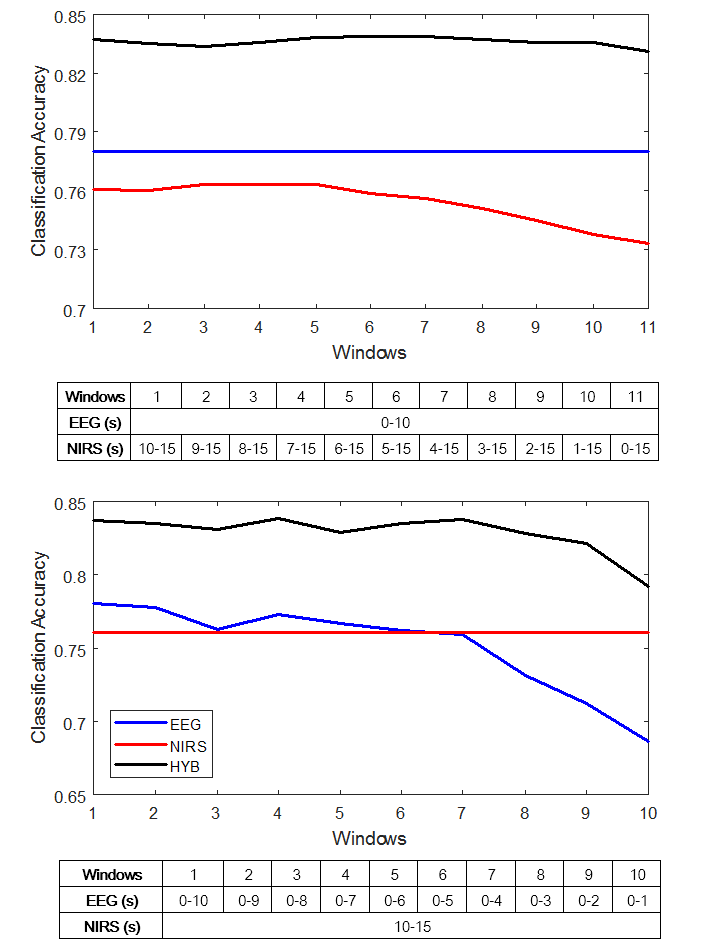


S1 Fig. Impact of analysis time window on classification performance. EEG, NIRS and HYB classification accuracies calculated by using various time windows. The Tables below the figures denote time periods for EEG and NIRS data used for calculating the corresponding classification accuracies. For the upper panel, the EEG time window is fixed and the NIRS time window varies, while the EEG time window varies and the NIRS time window is fixed for the lower panel.
